# A major pleiotropic QTL identified for yield components and nitrogen content in rice (*Oryza sativa* L.) under differential nitrogen field conditions

**DOI:** 10.1371/journal.pone.0240854

**Published:** 2020-10-20

**Authors:** T. Vishnukiran, C. N. Neeraja, V. Jaldhani, P. Vijayalakshmi, P. Raghuveer Rao, D. Subrahmanyam, S. R. Voleti

**Affiliations:** ICAR-Indian Institute of Rice Research, Rajendranagar, Hyderabad, India; Bahauddin Zakariya University, PAKISTAN

## Abstract

To identify the genomic regions for yield and NUE of rice genotypes and lines with promising yield under low N, a recombinant inbred population (RIL) developed between BPT5204 (a mega variety known for its quality) and PTB1 (variety with high NUE) was evaluated for consecutive wet and dry seasons under low nitrogen (LN) and recommended nitrogen (RN) field conditions. A set of 291 RILs were characterized for 24 traits related to leaf, agro-morphological, yield, N content and nitrogen use efficiency indices. More than 50 RILs were found promising with grain yield >10 g under LN. Parental polymorphism survey with 297 SSRs and selective genotyping revealed five genomic regions associated with yield under LN, which were further saturated with polymorphic SSRs. Thirteen promising SSRs were identified out of 144 marker trait associations under LN using single marker analysis. Composite interval mapping showed 37 QTL under LN with five pleiotropic QTL. A major stable pleiotropic (RM13201—RM13209) from PTB1 spanning 825.4 kb region associated with straw N % (SNP) in both treatments across seasons and yield and yield related traits in WS appears to be promising for the MAS. Another major QTL (RM13181-RM13201) was found to be associated with only relative trait parameters of biomass, grain and grain nitrogen. These two major pleiotropic QTL (RM13201-RM13209 and RM13181-RM13201) on chromosome 2 were characterized for their positive allele effect and could be deployed for the development of rice varieties with NUE.

## Introduction

Rice is one of the main cereal crops and staple food for more than half of the world population. Despite the increase in world rice production since the past half century, rice yield improvement is still required to support the growing needs of the increasing world population. Nitrogen (N) is the key nutrient required in large quantities for rice production [1]. Nearly 60% of the applied N is lost to the environment through combinations of different processes causing negative effects to the climate [2, 3]. Therefore, selection of rice genotypes with efficiency in N usage / low nitrogen (LN) tolerance is important in achieving better N utilization with minimum negative impact on environment and reducing the cost of cultivation [4]. Genetic variation in grain yield in relation to nitrogen is well reported in rice [5–8]. Grain yield of rice is a complex agronomic trait which is dependent on number of panicles, number of grains and grain weight [9]. Number of panicles per plant is in turn dependent on tillering ability of the genotype based on the dosage of N fertilization. Path coefficient analysis identifies contribution of each character to the trait of interest based on portioning of the correlation coefficients into direct and indirect effects and it was used in identifying the contribution of various components to yield in rice by Chandra et al. [10]; Cyprien and Kumar [11]; Seyoum et al. [12].

Mapping of the genomic regions associated with nitrogen use efficiency (NUE) would be useful for marker assisted selection and introgression into popular rice varieties. Several QTL have been reported in rice for various agro- morphological and yield characters under low N [13]. Genomic regions for plant height were identified under two N levels in doubled haploid (DH) lines by Fang and Wu et al. [14]. Tong et al. [15] detected 15 QTL in chromosomal segment substitution lines (CSSLs) under low N conditions. Seventeen QTL for agronomic, yield traits and N concentration characters in recombinant inbred lines (RILs) were identified under low and normal N [16]. Twenty three QTL for different morphological, agronomic, NUE and yield traits were identified in DH lines under native, optimum and high N [17]. Similarly, Tong et al. [18] identified seven QTL for yield traits in RILs under low, medium and high N. Eighteen QTL for yield components were identified in RILs under low and normal N [19]. Five QTL for plant height, tillers and NUE were identified in RILs under low N by Nguyen et al. [20]. Ogawa et al. [21] identified eight QTL in CSSLs under two N levels (native N, farmers practice N). Using Single Nucleotide Polymorphic (SNP) markers, several QTL have been identified for nutrient use efficiency traits by Jewel et al. [22] and Mahender et al. [23].

With an objective of identifying QTL for yield, nitrogen and their related traits and promising lines under low N, a RIL population was developed from two rice varieties with significant differences for yield under low N *viz*., BPT5204 and PTB1. The two parents and their RILs were evaluated under low N as well as recommended N for leaf, agro-morphological, yield and yield related traits, N content and N use efficiency indices,. Genomic regions/QTL for target traits and parameters of the study were identified through single marker analysis, selective genotyping and Composite Interval Mapping (CIM) method.

## Material and methods

### Field evaluation of mapping population

Based on preliminary screening of 107 rice genotypes under low and recommended N field conditions, two parents *viz*., BPT5204 and PTB1 were selected (S1 Table) [24]. BPT5204 is a mega rice variety known for its quality was released by Acharya NG Ranga Agricultural University, Bapatla, Andhra Pradesh, India) and PTB1 was a released rice variety from Regional Agricultural Research Station, Pattambi, Kerala, India. Mapping population of 291 RILs was developed from the parents BPT5204 and PTB1 by single seed descent method. During 2014 (WS) and 2015 (DS), RILs along with parental lines were grown at ICAR-Indian Institute of Rice Research farm (IIRR), Rajendranagar, Hyderabad. Two separate plots were maintained with zero nitrogen application (LN) and recommended nitrogen (RN) since 2011 at IIRR as described by Vijayalakshmi et al. [7]. Soil properties of the two plots (S2A Table) and weather parameters (S2B Table) during the experiment were recorded. Recommended package of rice crop production and protection practices were followed (www.rkmp.co.in). A set of 54 genotypes was also grown under LN and RN during 2014 (WS) and 2015 (DS) (S3 Table).

#### Phenotyping

Leaf length (cm) (LL), leaf width (cm) (LW) and leaf area (cm) (LA) were measured according to Yoshida et al. [25]. *In situ* leaf chlorophyll content (SPAD) was recorded with Minolta Corporation’s Chlorophyll SPAD-502 plus, USA. Three observations from each leaf at three positions were taken in three replications and average was reported. Days to 50% flowering (DFF) of each RIL was noted. At physiological maturity, plant height (cm) (PH), number of tillers (TNO), panicles per plant (PNO), grain yield (g/plant) (GY), hundred seed weight (g) (HSW), grain number per plant (GRNO) and total dry matter (g/plant) (TDM) were recorded from five plants of parents and RILs. Nitrogen percentage in grain (GNP), straw (SNP), total grain nitrogen per plant (GNPP) and straw nitrogen per plant (SNPP) were estimated following Kjeldahl method and nitrogen harvest index (NHI) was calculated. Physiological nitrogen use efficiency (PNUE), agronomic nitrogen use efficiency (ANUE) and agro-physiological efficiency (APE) were determined according to Fageria et al. [26]. Nitrogen deficiency tolerance traits *viz*., relative grain yield (RGY), relative biomass yield (RBM), relative grain nitrogen (RGN) and relative biomass nitrogen (RBN) were calculated according to Wei et al. [27]. Data for grain yield (g/plant) (GY) was noted for 54 genotypes in 2014 (WS) and 2015 (DS) (S3 Table).

### Genotyping

**Fig 1:**
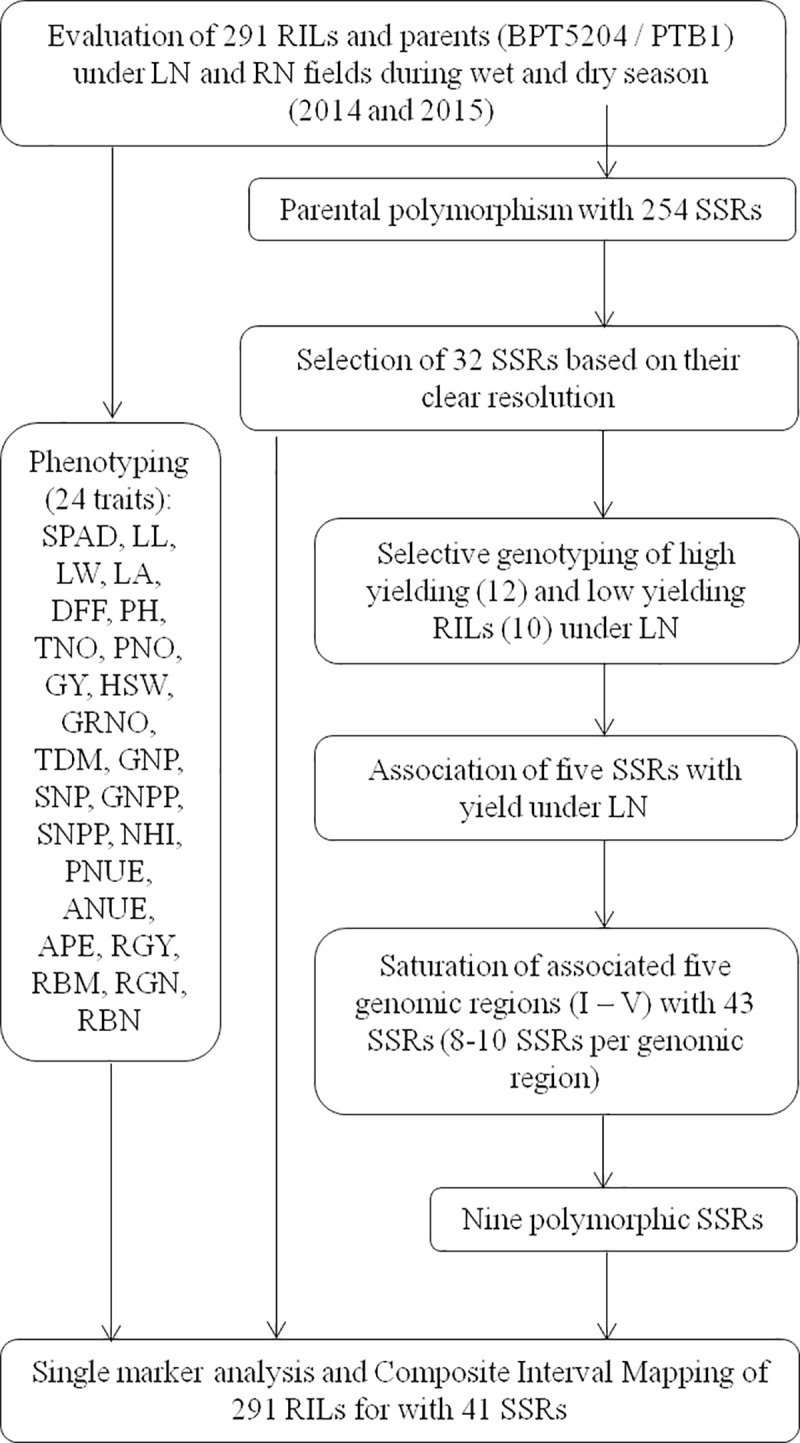
Schematic outline of phenotyping, parental polymorphism survey, selective genotyping, local linkage map construction and mapping. SPAD: SPAD value, LL: leaf length (cm), LW: leaf width (cm), LA: leaf area (cm), DFF: days to 50% flowering, PH: plant height (cm), TNO: tiller number, PNO: panicle number: GRNO: grain number, HSW: hundred seed weight (g), GY: grain yield per plant (g/plant), TDM: total dry matter (g/plant), GNP: grain nitrogen percent, SNP: straw nitrogen percent, GNPP: grain nitrogen/ plant, SNPP: straw nitrogen/ plant, NHI: nitrogen harvest index, RGY: relative grain yield, RBM: relative biomass yield, RGN: relative grain nitrogen, RBN: relative biomass nitrogen, PNUE: physiological nitrogen use efficiency, ANUE: agronomic nitrogen use efficiency, APE: agro- physiological efficiency.

DNA from the fresh leaf tissues of parents and RILs was isolated according to modified protocol of Zheng et al. [28]. Parental polymorphism was surveyed with 254 rice microsatellite (RM) or simple sequence repeats (SSR) markers from Gramene database (http://www.gramene.org). The details of SSRs and their status of polymorphism were given in S4 Table. The schematic outline of mapping is presented in Fig 1.

For amplification of the SSRs, PCR was carried out following the procedure described by Rao et al. [29] with 15μl reaction using Veriti thermal cycler (Applied Biosystems). The amplified products were resolved with 3% agarose gel (Lonza, USA) and gels were stained with 0.5 mg/ml ethidium bromide and bands were visualized using Alpha Imager 1220 (Alpha Innotech, USA). Selective genotyping was performed with 32 polymorphic SSRs in two groups of RILs comprising 12 RILs with highest grain yield > 10 g and 10 RILs <1 g with the lowest yield under LN. The phenotypic and genotypic data of 22 RILs and parents was analyzed and local linkage maps were constructed using MapDisto v. 1.7 [30]. Five SSRs associated with grain yield from the selective genotyping analyses *viz*., RM1, RM7075, RM9, RM13021 and RM13197 were considered as anchor SSRs. Eight to 10 SSRs spanning each associated anchor SSR were selected from Gramene database for parental polymorphism and the nine polymorphic SSRs were screened in RILs.

### Data analysis

Two way Analysis of variance (ANOVA) was performed for the phenotypic data using open software R language [31] with agricolae package [32] and statistical significance was determined for parents and RILs. Path coefficient analysis was performed using correlation values according to Dewey and Lu [33] and direct and indirect effects were calculated [34].

### Single marker analysis (SMA)

The phenotypic data of 291 RILs and genotypic data of 41 (32+9) polymorphic SSRs was subjected to SMA using ANOVA of open software R [32].

### Composite interval mapping (CIM)

The linkage maps for chromosomes 1 and 2 spanning polymorphic SSRs in five anchor regions were constructed using Mapmaker and were imported into QTL Cartographer (Win QTL Cart 2.5). Composite interval mapping (CIM) was performed with kosambi function for 25 SSRs (chromosome 1: 15; chromosome 2: 10) and QTL were identified with minimum LOD score of 2.5 with 1000 permutations.

### Allelic effect of associated SSRs

The average allelic effect (AAE) of two SSRs (RM13209 and RM13181) from two major QTL viz., RM13201-RM13209 and RM13181-RM13201 was estimated by comparing the mean phenotypic data for that trait with respect to each allele to the phenotypic data of the null allele (a_i_ = phenotypic effect of the allele i = ∑x_ij_/n_i_—∑N_k_/n_k_; x_ij_ = phenotypic measurement values of j genotype carrying the allele i; n_i_ = the number of genotypes carrying the allele of i, N_k_ = phenotypic value of the genotype of k carrying null allele, n_k_ = the number of genotypes for the null allele [35].

### Co-localization

Positions of the associated SSRs identified through SMA and CIM of the present study were compared to the genomic positions of SSRs from the reported QTL to study co-localization. The associated SSRs and QTL under LN were also compared with the reported QTL of normal N (recommended N). The positions of flanking SSRs of genomic regions associated with two or more than two QTL under LN were retrieved from https://blast.ncbi.nlm.nih.gov/Blast.cgi and analyzed for the putative candidate genes in the genomic region of QTL.

## Results

### Phenotyping

Significant variation was observed between the parents (BPT5204 and PTB1) for 24 phenotype traits/parameters studied under LN (S5 Table). Substantial variability was also noted for 24 traits/parameters between treatments and among the RILs under LN and RN across seasons (Tables 1 and 2). There was overall reduction for the 24 traits/parameters of the study under LN in comparison to RN for both parents, however the reduction of relatively less in PTB1 (better NUE) than BPT5204 (S6 Table). The magnitude of reduction between the parents and RILs was observed to be varying across seasons. The mean relative grain yield, biomass yield and relative grain and biomass nitrogen was higher in PTB1, whereas physiological, agronomical and agro-physiological nitrogen use efficiencies were higher in BPT5204. Transgressive variants for yield in RILs were observed under LN (Tables 1 and 2). Thirty nine RILs in WS and 14 RILs in DS were found promising with >10 g under LN.

**Table 1:** ANOVA for leaf, agro-morphological, yield and yield related traits and N content and N use efficiency indices of parents and RILs in wet season.

Traits/Parameters	RILs				BPT5204		PTB1		ANOVA			
	Range		Mean						Among RILs			
	Low N	Rec N	Low N	Rec N	Low N	Rec N	Low N	Rec N	Low N	Rec N	Treatment	RILs X Treatment
LL	12.7–32.6	18.25–46.6	22.3	29.7	21.0	28.0	22.8	25.0	***	***	***	*
LW	0.47–1.1	0.73–1.4	0.81	0.97	1.2	1.4	0.9	1.1	***	***	***	*
LA	6.04–22.74	11.62–41.04	13.67	21.77	18.90	28.70	14.81	20.63	***	***	***	**
SPAD	22–40.9	29.8–47.1	32.6	37.1	38.9	53.8	44.1	52.0	***	***	***	*
DFF	95–122	100–132	105	109	112	131	107	110	***	***	***	^NS^
PH	77–131	88.5–153	99.5	118.6	57.7	70.3	100.0	103.7	***	***	***	***
TNO	2–14.0	4–20.0	5	8	5	16	5	13	***	***	***	***
PNO	2–11.0	3–19.0	4	7	5	16	4	10	***	***	***	**
GY	1.40–28.7	4.37–30.67	6.96	12.93	3.89	16.37	6.09	18.13	***	***	***	***
HSW	0.95–2.47	1.14–2.61	1.62	1.78	1.04	1.04	1.85	1.94	***	***	***	^NS^
GRNO	97–1712	239–2990	440	737	374	709	333	933	***	***	***	**
TDM	7.73–68.53	15.08–112.6	18.64	31.40	10.72	25.33	26.03	30.67	***	***	***	***
GNP	0.15–1.45	0.75–1.76	0.83	1.28	0.94	1.39	1.06	1.49	***	***	***	^NS^
SNP	0.01–0.58	0.08–1.23	0.20	0.41	0.51	0.83	0.56	1.00	***	***	***	*
GNPP	0.01–0.44	0.05–0.84	0.07	0.19	0.04	0.10	0.06	0.27	***	***	***	*
SNPP	0.01–3.62	0.01–0.96	0.09	0.39	0.04	0.15	1.72	0.13	***	***	***	^NS^
NHI	33.55–99.42	46.08–94.82	80.49	76.41	64.86	62.56	65.29	59.70	***	***	***	^NS^
PNUE	7.06–130.01		45.09		57.74		11.5		***			^ ^
ANUE	1.09–114.94		25.05		14.62		50.6		***			^ ^
APE	1.52–63.6		27.71		16.49		37.61		***			^ ^
RGY	8.39–97.43		56.59		52.99		35.16		***			^ ^
RBM	19.72–92.15		62.77		42.48		84.96		***			^ ^
RGN	11.94–96		65.62		67.18		71.9		***			^ ^
RBN	0.81–89.03		50.3		61.25		56.37		***			^ ^

Significance at the level of 0.05 (*), 0.01 (**) and 0.001 (***). SPAD: SPAD value, LL: leaf length (cm), LW: leaf width (cm), LA: leaf area (cm), DFF: days to 50% flowering, PH: plant height (cm), TNO: tiller number, PNO: panicle number: GRNO: grain number, HSW: hundred seed weight (g), GY: grain yield per plant (g/plant), TDM: total dry matter (g/plant), GNP: grain nitrogen percent, SNP: straw nitrogen percent, GNPP: grain nitrogen/ plant, SNPP: straw nitrogen/ plant, NHI: nitrogen harvest index, RGY: relative grain yield, RBM: relative biomass yield, RGN: relative grain nitrogen, RBN: relative biomass nitrogen, PNUE: physiological nitrogen use efficiency, ANUE: agronomic nitrogen use efficiency, APE: agro-physiological efficiency.

**Table 2:** ANOVA for leaf, agro-morphological, yield and yield related, N content and N use efficiency indices of parents and RILs in dry season.

Traits/Parameters	RILs				BPT5204		PTB1		ANOVA			
	Range		Mean						Among RILs			
	Low N	Rec N	Low N	Rec N	Low N	Rec N	Low N	Rec N	Low N	Rec N	Treatment	RILs X Treatment
LL	12.4–35.1	19–46.2	21.6	28.5	17.6	21.0	21.2	22.1	***	***	***	^NS^
LW	0.6–1.13	0.77–1.43	0.90	1.13	1.0	1.2	0.9	1.1	***	***	***	^NS^
LA	6.68–26.35	12.8–46.01	14.7	24.29	13.18	19.44	14.29	18.26	***	***	***	^NS^
SPAD	25.9–41.7	31.8–46.2	35.1	39.3	38.0	38.8	41.1	41.9	***	***	***	**
DFF	99–132	108–143	116	127	116	119	102	112	***	***	***	^NS^
PH	67.7–124	79.3–148.7	94.9	117.5	57.7	69.7	97.3	133.3	***	***	***	***
TNO	3–12.0	7–21.0	6	10	6	12	10	12	***	***	***	***
PNO	2–12.0	6–18.0	6	10	6	12	10	12	***	***	***	***
GY	0.5–15.3	2.90–34.4	5.94	13.59	2.07	16.43	4.57	15.67	***	***	***	**
HSW	0.69–2.32	1.09–2.57	1.47	1.70	1.90	2.03	1.05	1.19	***	***	***	^NS^
GRNO	29–1055	210–2006	416	818	110	1106	434	994	***	***	***	*
TDM	7.1–42.73	11.33–74.27	17.90	34.60	6.60	25.87	14.07	29.63	***	***	***	**
GNP	0.51–1.60	1.02–1.73	1.05	1.36	0.58	1.10	1.34	1.46	***	***	***	***
SNP	0.02–0.62	0.13–1.09	0.19	0.35	0.39	0.97	0.89	1.13	***	***	***	^NS^
GNPP	0.01–0.21	0.06–0.64	0.07	0.21	0.01	0.25	0.06	0.13	***	***	***	*
SNPP	0.002–0.11	0.01–0.43	0.03	0.09	0.02	0.03	0.09	0.20	***	***	***	^NS^
NHI	54.66–98.06	58.26–92.04	85.05	80.31	59.91	53.20	60.55	50.16	***	***	***	^NS^
PNUE	15.57–165.11		82.08		76.56		84.52		*			
ANUE	4.89–96.32		32.5		85.53		29.82		***			
APE	2.76–86.45		36.94		86.45		38.86		^NS^			
RGY	2.67–87.53		47.29		9.5		39.61		***			
RBM	15.21–91.76		55.86		26.86		47.56		***			
RGN	34.99–118.79		77.49		53.06		92.16		***			
RBN	3.01–88.36		55.01		40.37		78.54		***			

Significance at the level of 0.05 (*), 0.01 (**) and 0.001 (***). SPAD: SPAD value, LL: leaf length (cm), LW: leaf width (cm), LA: leaf area (cm), DFF: days to 50% flowering, PH: plant height (cm), TNO: tiller number, PNO: panicle number: GRNO: grain number, HSW: hundred seed weight (g), GY: grain yield per plant (g/plant), TDM: total dry matter (g/plant), GNP: grain nitrogen percent, SNP: straw nitrogen percent, GNPP: grain nitrogen/ plant, SNPP: straw nitrogen/ plant, NHI: nitrogen harvest index, RGY: relative grain yield, RBM: relative biomass yield, RGN: relative grain nitrogen, RBN: relative biomass nitrogen, PNUE: physiological nitrogen use efficiency, ANUE: agronomic nitrogen use efficiency, APE: agro-physiological efficiency.

### Correlation and path analysis

**Fig 2:**
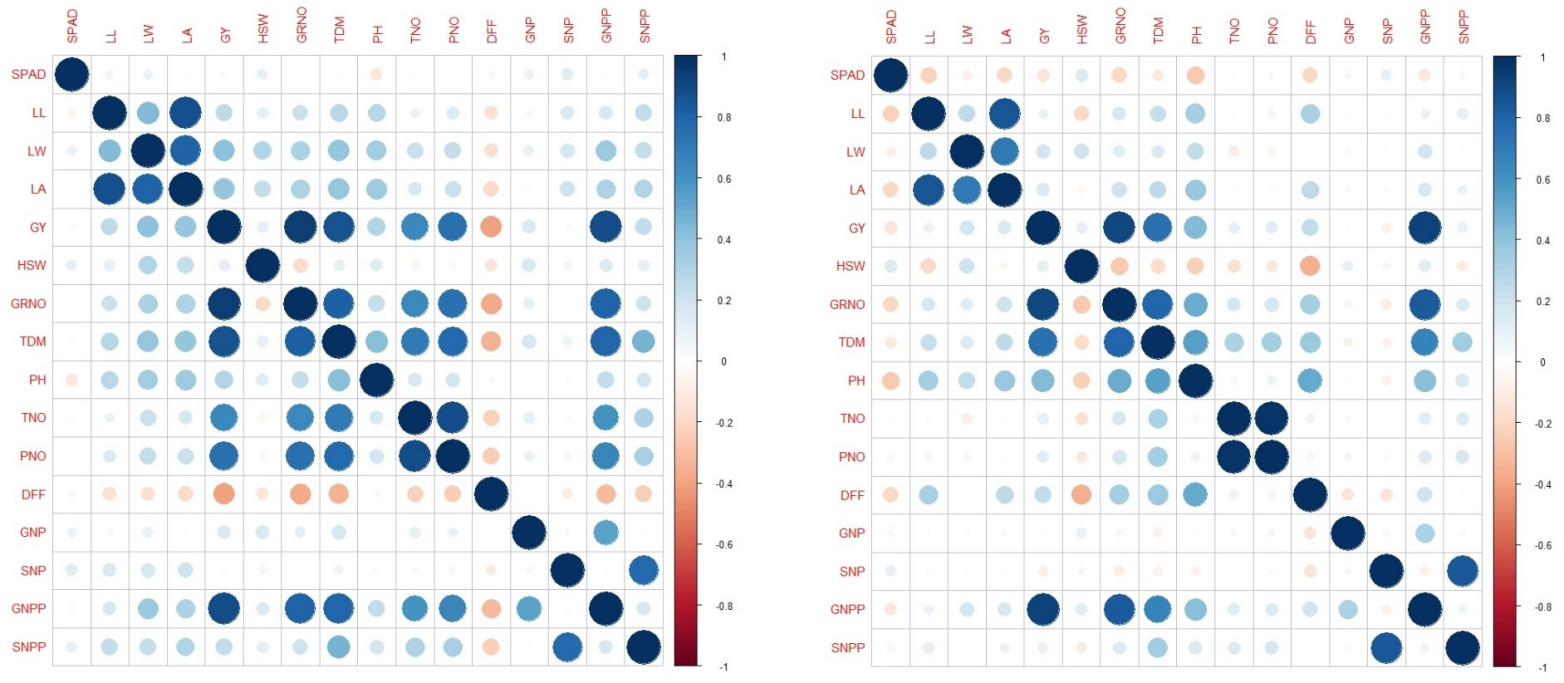
**A.** Correlation matrix under low N in wet season; **B.** Correlation matrix under low N in dry season. SPAD: SPAD value, LL: leaf length (cm), LW: leaf width (cm), LA: leaf area (cm), DFF: days to 50% flowering, PH: plant height (cm), TNO: tiller number, PNO: panicle number: GRNO: grain number, HSW: hundred seed weight (g), GY: grain yield per plant (g/plant), TDM: total dry matter (g/plant), GNP: grain nitrogen percent, SNP: straw nitrogen percent, GNPP: grain nitrogen/ plant, SNPP: straw nitrogen/ plant.

Under LN, GY was found to be positively correlated with PH, TNO, PNO, GRNO, TDM and GNPP across seasons as expected. Association of GY with PH was observed exclusively under LN across seasons (Fig 2A and 2B). Significant positive correlations were observed between PNUE, ANUE and APE with GY only under RN (S1A–S1D Fig). Path coefficient analysis revealed 20 significant positive direct effects on GY under LN. Among them, 12 direct positive effects with LL, TNO, PNO, GNP, SNPP in WS, eight with SPAD in DS and seven traits (LW, LA, GRNO, TDM, PH, PNO, GNPP) across seasons were identified (S7A and S7B Table). Only PH has shown exclusive positive direct effect under LN. Under RN, seven traits (LW, GRNO, TDM, TNO, PNO, GNPP, and SNPP) were common across seasons (S7C and S7D Table). Five traits were common across RN and LN in WS (DFF, GRNO, TDM, PNO, and GNPP) and three traits were common in DS (GRNO, TDM, GNPP).

### Parental polymorphism and selective genotyping

**Fig 3:**
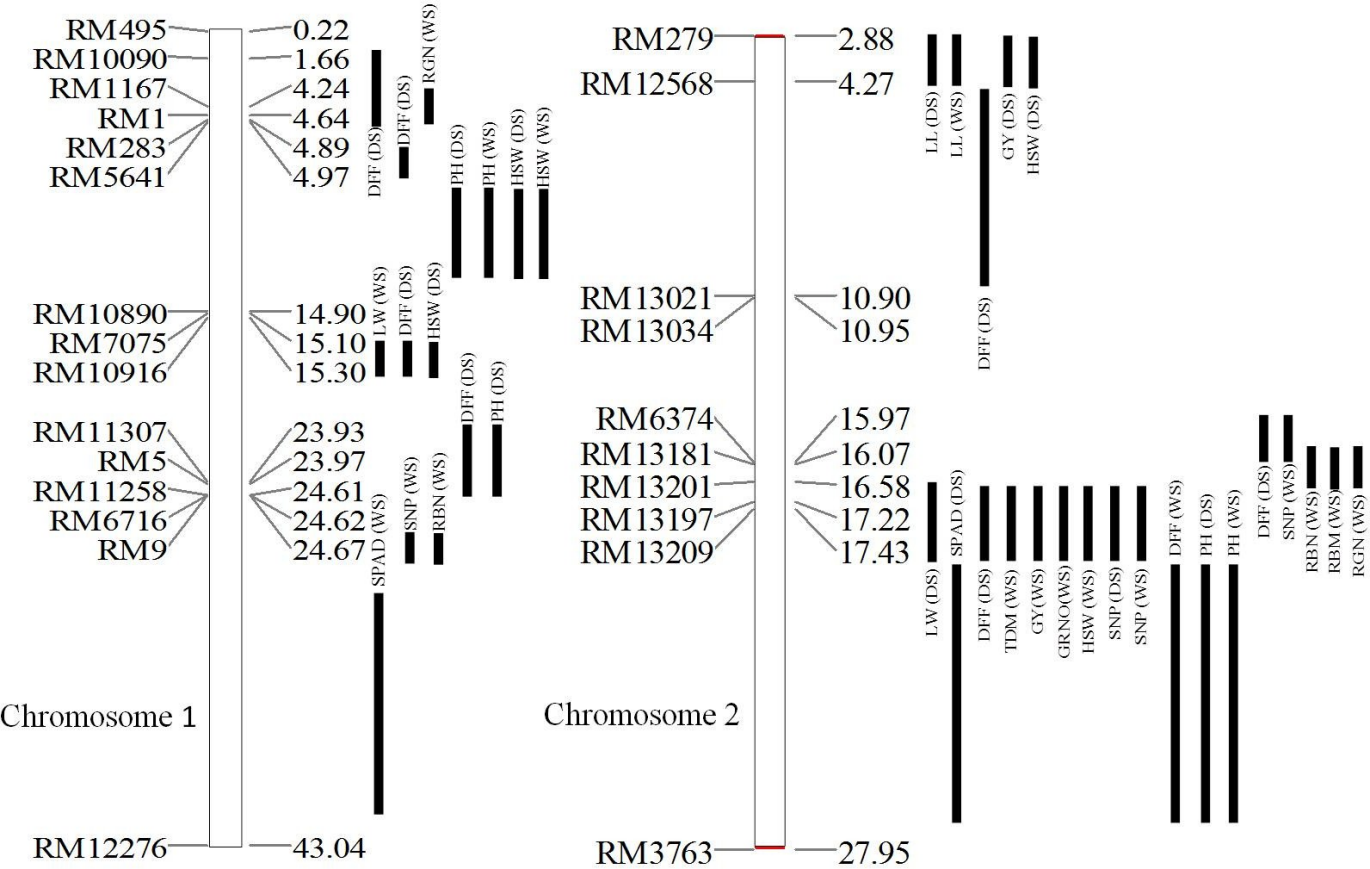
Local linkage map of chromosomes 1 and 2 with identified QTL under LN. WS: Wet season, DS: Dry season, SPAD: SPAD value, LL: leaf length (cm), LW: leaf width (cm), DFF: days to 50% flowering, PH: plant height (cm), GRNO: grain number, HSW: hundred seed weight (g), GY: grain yield per plant (g/plant), TDM: total dry matter (g/plant), SNP: straw nitrogen percent, RBM: relative biomass yield, RGN: relative grain nitrogen, RBN: relative biomass nitrogen.

Between the two parents, 42 SSRs showed polymorphism (16.5%) out of 254 SSRs and 32 SSRs were selected for further analyses based on their resolution. Yield being identified as the important selection criterion under LN, selective genotyping of 22 RILs (12 RILs with highest grain yield and 10 RILs with the lowest yield under LN) was performed. Five SSRs have shown association with grain yield under LN using MapDisto (S8 Table). Among the five SSRs identified as anchor markers, three SSRs were on chromosome 1 *viz*., genomic region I—RM1, genomic region II—RM7075, genomic region III—RM9 and two SSRs on chromosome 2 (genomic region IV—RM13021 and genomic region V—RM13197) (Fig 3).

#### Saturation of identified grain yield associated genomic regions with SSRs

Screening of the five associated genomic regions with markers flanking the anchor SSR has shown a total of nine polymorphic SSRs *vi*z., one at genomic region I, two at genomic region II, two at genomic region III, one at genomic region IV and three at genomic region V (S9 Table).

### Single marker analysis (SMA)

SMA showed 144 marker trait associations with 31 SSRs (trait associations were not found with RM283) across seasons under LN for 22 traits (marker associations were not found for SNP and RBN) (S10A and S10B Table). More number of marker trait associations was observed during DS. Only seven trait marker associations were detected in common across seasons exclusively under LN (S2 Fig). Maximum number of marker associations (16) was noted for GNPP. RM5455 (chromosome 7) was found to be associated with maximum number of traits (14). SMA for nine polymorphic SSRs associated with yield through selective genotyping also revealed 45 associations with 24 traits are presented trait wise (S11A Table) and marker wise (S11B Table). Leaf length was associated with three SSRs in WS, one in DS and two SSRs (RM10090, RM1189) were detected across seasons in common. Six marker trait associations observed for LW with four SSRs exclusively in DS and two common SSRs (RM7075 and RM13197) across seasons. LA showed six marker trait associations with RM1189 common across seasons. For SPAD, three SSRs (RM7075, RM15855, and RM7110) were identified in common; four SSRs were found in DS and one SSR in WS. A total of three SSRs (RM10090, RM3763, and RM1189) were associated with DFF across seasons, with two SSRs associated in WS and three SSRs associated in DS. For PH, two common SSRs (RM20350, RM20746) were found across seasons and five SSRs in DS, while marker trait association was not found in WS. For TNO, one SSR (RM5543) was found in common across seasons with two SSRs in WS and three SSRs in DS. For PNO, common marker trait association was not observed across seasons, but three SSRs each were identified in WS and DS. For GY, only one SSR (RM19341) has shown association in common across seasons and four SSRs in WS and five SSRs in DS were found to be associated. For GRNO, RM20746 was detected in common across seasons and two SSRs were associated in DS without any marker trait association in WS. For HSW, common marker trait association was not found across seasons, however RM24829 was detected in WS and three SSRs were identified in DS. For TDM, RM13197 has shown common association across seasons with associations of one SSR in WS and 10 SSRs in DS. For GNP, a common SSR (RM13197) was detected across seasons and four SSRs in WS and three SSRs in DS were found. Marker trait association was not found for SNP. Three common SSRs (RM5, RM11307, and RM19341) were identified for GNPP, while association of six SSRs in WS and association of seven SSRs in DS were observed. While common marker was not found across seasons for SNPP, association of RM495 in WS and two SSRs in DS were identified. For NHI, four SSR associations were observed only in WS, while marker trait associations were not observed either in common or in DS. Among seven NUE indices, only RM13197 and RM171 showed association in common across season for RGN and RGY. During WS, four SSRs with ANUE and RGN, three SSRs with APE, two SSRs with PNUE, one SSR each with RBM and RGY were found to be associated. Association of four SSRs each with APE and RBM, two SSRs each with ANUE and RGN, six SSRs with RGY were detected in DS. The trait associations of nine polymorphic SSRs of the five anchor marker regions were presented marker wise (S11 Table).

### Composite interval mapping (CIM)

CIM identified 37 QTL under LN (Table 3) and 32 QTL under RN for chromosomes 1 and 2 (S12 Table). Among the tested traits, maximum number of QTL were identified for DFF (8) followed by HSW (5) and PH (5 QTL) and SNP (4 QTL) under LN.

**Table 3:** Details of identified QTL for leaf, agro-morphological, yield and yield related traits and N content and its indices identified through CIM under low N in WS and DS.

SNO	Season	Treat	Trait	Chromosome	Position(cM)	Marker Interval	LOD	R2
1	Dry	Low	LL	2	4.01	RM279—RM12568	2.50	5.32
2	Wet	Low	LL	2	14.01	RM279—RM12568	3.16	8.53
3	Wet	Low	LW	1	759.51	RM7075—RM10916	2.51	10.85
4	Dry	Low	LW	2	573.41	RM13201—RM13209	3.24	5.24
5	Wet	Low	SPAD	1	1122.61	RM9—RM12276	2.81	9.68
6	Dry	Low	SPAD	2	653.71	RM13209—RM3763	4.39	14.22
7	Dry	Low	DFF	1	330.21	RM10090—RM1	7.47	63.49
8	Dry	Low	DFF	1	923.61	RM11307—RM11258	12.30	66.90
9	Dry	Low	DFF	1	637.61	RM283—RM5641	7.38	63.48
10	Dry	Low	DFF	1	776.51	RM7075—RM10916	7.73	64.42
11	Dry	Low	DFF	2	597.51	RM13201—RM13209	10.97	60.66
12	Dry	Low	DFF	2	47.71	RM12568 –RM 13021	8.86	56.66
13	Dry	Low	DFF	2	354.31	RM6374—RM13181	8.14	55.17
14	Wet	Low	DFF	2	656.71	RM13209—RM3763	2.99	9.12
15	Dry	Low	PH	1	880.51	RM11307—RM11258	5.77	37.19
16	Dry	Low	PH	1	721.61	RM5641—RM10890	3.53	26.40
17	Wet	Low	PH	1	704.61	RM5641—RM10890	2.68	3.95
18	Wet	Low	PH	2	653.71	RM13209—RM3763	7.30	27.51
19	Dry	Low	PH	2	651.71	RM13209—RM3763	3.94	17.35
20	Wet	Low	TDM	2	583.51	RM13201—RM13209	7.59	57.03
21	Dry	Low	GY	2	2.01	RM279—RM12568	3.46	6.08
22	Wet	Low	GY	2	581.51	RM13201—RM13209	7.92	43.38
23	Wet	Low	GRNO	2	582.51	RM13201—RM13209	4.22	38.19
24	Dry	Low	HSW	1	710.61	RM5641—RM10890	3.31	8.49
25	Dry	Low	HSW	1	757.51	RM7075—RM10916	6.10	27.67
26	Wet	Low	HSW	1	705.61	RM5641—RM10890	3.77	5.88
27	Dry	Low	HSW	2	11.01	RM279—RM12568	6.49	14.82
28	Wet	Low	HSW	2	573.41	RM13201—RM13209	12.74	17.70
29	Dry	Low	SNP	2	585.51	RM13201—RM13209	3.53	47.65
30	Wet	Low	SNP	2	595.51	RM13201—RM13209	3.21	48.62
31	Wet	Low	SNP	1	937.61	RM6716—RM9	2.53	57.52
32	Wet	Low	SNP	2	347.31	RM6374—RM13181	2.64	53.07
33	Wet	Eff	RBN	1	939.61	RM6716—RM9	4.70	59.43
34	Wet	Eff	RBN	2	499.41	RM13181—RM13201	4.42	60.75
35	Wet	Eff	RBM	2	433.41	RM13181—RM13201	2.73	56.23
36	Wet	Eff	RGN	1	344.21	RM1167—RM1	2.73	56.53
37	Wet	Eff	RGN	2	444.41	RM13181—RM13201	2.71	58.02

SPAD: SPAD value, LL: leaf length (cm), LW: leaf width (cm), DFF: days to 50% flowering, PH: plant height (cm), GRNO: grain number, HSW: hundred seed weight (g), GY: grain yield per plant (g/plant), TDM: total dry matter (g/plant), SNP: straw nitrogen percent, RBM: relative biomass yield, RGN: relative grain nitrogen, RBN: relative biomass nitrogen.

#### Leaf traits

Four QTL were identified for leaf length (LL) with two QTL under LN and two QTL under RN. QTL with marker interval of RM279—RM12568 was identified in both WS and DS under LN. A total of four QTL were detected for leaf width (LW) with two QTL each under LN and RN. Four QTL were identified only under RN for leaf area (LA) with two each in WS and DS. There were no QTL identified for LA under LN. Under LN, two QTL are identified for LA with one each in WS and DS.

#### Yield and yield related traits

Four QTL were identified for grain yield (GY)/plant with two QTL each under LN and RN. Under LN, a major QTL with marker interval of RM13201—RM13209 (WS) (PV 43.3%) and a minor QTL (RM279—RM12568) during DS were identified. A total of 10 QTL were detected for hundred seed weight (HSW), with five each under LN and RN. Under LN, a common QTL was identified in WS and DS with marker interval of RM5641—RM10890. One QTL was detected for grain number (GRNO) under RN.

#### Agro-morphological traits

A total of three QTL were detected for total dry matter (TDM)/plant under LN (1 QTL) and RN (2 QTL). A total of seven QTL were detected for plant height (PH) under LN (5 QTL) and RN (2 QTL) with two QTL detected in common across seasons under LN. One QTL was detected tiller number (TNO) under RN and QTL were not detected under LN.A total of 14 QTL were detected for days for 50% flowering (DFF) with eight QTL under LN and six QTL under RN.

#### N content and its indices

A total of seven QTL were detected for straw N % (SNP) with four QTL under LN and three QTL under RN. Under LN, one major QTL (PV >48%) was detected for SNP across seasons with marker interval of RM13201—RM13209. Two major QTL for relative grain N (RGN) and another two major QTL for relative biomass N (RBN) on chromosomes 1 and 2 were detected. One major QTL for relative biomass yield (RBM) shared its location with QTL for RGN and RBN.

### Co-localization of associated genomic regions and markers of the present study with the reported QTL

The identified QTL for the chromosomes 1 and 2 co-localized with positions of reported for similar trait QTL were presented trait wise in detail. The co-localization of associated SSRs with the reported QTL regions was also presented.

#### Yield and yield related traits

With reference to chromosome 1, RM10090, RM1167 and RM5641 associated with HSW under LN, co-localized with genomic region for thousand grain weight under LN [36] and RN [37]. Interestingly, the identified genomic region for HSW under LN and RN in DS (RM10890-RM10916) also shared its location with reported grain weight QTL *viz*., gw-1 and *qTGW 1–1* [38, 39]. Another QTL in the marker interval of RM11307-RM11258 matched with reported QTL for yield and yield related traits *viz*., grain weight, grain number per plant, spikelet number per plant and spikelets per panicle [39–41] in this region under drought stress conditions. RM12276 was associated with GY, GRNO and TDM under LN concurred with the reported genomic regions for spikelet fertility [42, 43].

For chromosome 2, genomic region for HSW (RM13021-RM13034) in the present study corresponded with reported QTL for number of grains per panicle (*qGN 2*), hundred grain weight (*qHW 2*) and grain density (*qGD 2*) [44]. GRNO markers (RM13021-RM13029) matched with the QTL for number of filled grains [45], number of spikelets per plant [46–48]. RM279 associated with GY under LN and RN in DS shared its location with genomic regions for yield under LN and RN [36]. RM3763 associated with HSW, PH and SPAD under both treatments and seasons co-localized with the reported genomic region for thousand grain weight [39, 49]. The SSRs associated with GY and GRNO (RM1167 and RM1) coincided with the previously reported genomic regions for yield and yield components such as spikelet number and panicle weight [50], yield per plant [51], spikelets for panicle [47, 52, 53], grain yield [54], spikelet density [55] under RN and grain number per panicle, spikelets per panicle under LN [20, 36]. Two associated SSRs on chromosome 3 have also shown concurrence with reported yield QTL. RM14250 (chromosome 3) associated with HSW under LN and RN shared its genomic region with the loci of thousand grain weight reported by Zhuang et al. [52], Zhang et al. [54], Fu et al. [56], Liu et al. [57]. RM15855 associated with GY under LN, corresponded with the QTL for grain weight and spikelet number per panicle [51, 57]. RM20746 of chromosome 5 was associated with HSW, GRNO, GY, GNPP, PH and TDM under LN overlapped with the reported genomic regions for yield/ plant [15], grain density/panicle [19], grain yield/plant [52] and grain weight [58]. Across chromosome 7, RM7110 associated with HSW, GRNO, TDM under LN corresponded with QTL for grain yield, spikelet number per panicle [36] and spikelet number per panicle and number of grains per panicle [56] under LN. Another SSR, RM5455 associated with HSW under both treatments and GRNO under LN has shared its locus with reported QTL for spikelets per panicle [18, 59, 60], thousand grain weight [59] and grain dimensions [61, 62]. RM20967 associated with GY and DFF in DS under low N corresponding with the QTL for 2^o^ rachis/panicle and biomass/plant [63]. The two SSR markers on chromosome 9 *viz*., RM1189 associated with HSW under LN, coincided with genomic region for thousand grain weight [60], grain thickness and width [61] and grain breadth [64]. RM24829 associated with HSW under LN, corresponded with the reported QTL for thousand grain weight [57]. RM287 on chromosome 11 was associated with HSW across the treatments and seasons and GY under LN matched with QTL regions reported for thousand grain weight [54], grain length, width and grain weight [58]. RM1159 on chromosome 12 associated with HSW under both LN and RN, concurred with QTL reported by Tong et al. [15] for grain weight. RM1159 (chromosome 12) associated with HSW under both treatments was matched with the reported region of grain weight QTL [15].

#### N content and N indices

The locations of RM11307, RM6716, RM11258 (chromosome 1) associated with RGY in DS corroborated with genomic regions for relative grain weight reported by Ogawa et al. [21]. Association of RM15855 (chromosome 3) with GNP, SPAD and PH under LN coincided with genomic region for photosynthetic ability, chlorophyll a/b ratio and plant height [65]. RM17201 (chromosome 4) was associated with GNPP and TDM under LN in WS and DS, concurred with genomic region for grain yield under LN [19]. Under LN, GNPP, GY, LL and LA were associated with RM19341 (chromosome 6) across seasons and this region corresponded with QTL location for flag leaf length, spikelet and grain number per panicle and grain weight [66].

#### Leaf length, width, area and SPAD

The SSR markers on chromosomes 1 and 2 (RM5641 and RM13021) associated with LL, LA and LW coincided with reported QTL for leaf traits [44, 47, 67–69]. In the present study, SPAD was associated with RM13034 (chromosome 2) under LN across seasons. This region was also identified for SPAD [46] and chlorophyll content under LN [15]. RM20350 (chromosome 6) associated with LL and SPAD under both treatments and across seasons, also corresponded to the reported genomic regions for flag leaf width, second leaf width and area [67] and region associated with stromal processing peptidase (*spp*) responsible for chloroplast biogenesis at early growth stage and root development [70]. RM22524 (chromosome 8) was associated with LL and LA under both treatments and seasons found to be coinciding with the QTL for flag leaf length, flag leaf width identified by Marathi et al. [47]. RM1189 (chromosome 9) associated with LL and LA under LN also found to be in the QTL region reported for flag leaf length [71].

#### Days for 50% flowering, plant height and number of tillers

Markers for DFF (RM10090, RM1) on chromosome 1 were also found to be coinciding with QTL location for days to heading [40, 48]. In the present study, DFF was associated under both LN (RM20746) and RN (RM19341) on chromosome 6 in DS also matched with region of QTL for heading date (*qhd-6*) identified by Bai et al. [60] and 50% flowering by Marathi et al. [47]. In the present study, genomic region for PH on chromosome 2 was detected by SMA (RM13197, RM13201, and RM13209) under LN and RN shared its location with genomic region for plant height (*qPHT 2–1*) as reported by Marathi et al. [47]. The SSRs identified for TNO (RM1, RM10090, RM11307, RM12276) of chromosome 1 under LN and RN in DS were found to be co-localizing with QTL locations for tiller number by Zang et al. [72] (*QTn1*) and Kaladhar et al. [40] (*nt 1*.*2*). RM7110 (chromosome 7) associated with PH and DFF under LN in DS has also co-localized with genomic regions for plant height and heading dates by Kotla et al. [73] and Liu et al. [74]. Similarly, PH and DFF in DS associated with RM1189 (chromosome 9) under LN shared its location with QTL identified for plant height [46, 71] and days to 50% flowering [75].

#### Positive average allele effect

On amplification of two SSRs (RM13201 and RM13181) from two major pleiotropic QTL (RM13201-RM13209 and RM13181-RM13201) in 54 genotypes, three alleles each were obtained per SSR. Two alleles (RM13029-255 bp and RM13181-270bp) same size as PTB1 have shown positive effect with grain yield (g) (Table 4).

**Table 4:** Positive average allele effect of RM13209 and RM13181 in 54 genotypes across seasons.

		WS	DS
Marker	Allele size (bp)	Grain yield/plant (g)	Grain yield/plant (g)
RM13209	255	0.492	0.528
RM13209	270	0.165	-0.412
RM13209	260	0.044	-0.590
RM13181	270	0.492	0.528
RM13181	280	0.165	-0.412
RM13181	260	0.044	-0.590

### In silico analysis for candidate gene identification

Out of the 37 QTL identified, five pleiotropic QTL affecting two or more than two traits were analyzed for the candidate genes *viz*., RM13201-RM13209 region spanning 825.4 kb region has 54 putative candidate genes; RM13181-RM13201 covering 467.706 kb has 42 candidate genes; RM5641-RM10890 spanning 9929.476 kb has 1207 candidate genes; RM7075-RM10916 covered 191.769 kb region with 27 candidate genes and RM279-RM12568 spanned 1392.02 kb region with 247 candidate genes (S13 Table).

## Discussion

Development of NUE rice varieties is important for utilizing N in the soil either applied or naturally available. The dose and application of N can be adjusted according to the requirement of NUE varieties. Thus lesser dose and application of N would reduce the N losses to the environment impacting climate change and also the cost of rice cultivation. The identification of QTL/genomic regions associated with yield and its related traits under LN or NUE could be deployed in marker-assisted selection for improving the NUE of rice. Several QTL have been reported for NUE and related traits/parameters in biparental mapping populations of rice and their deployment in the breeding program is scarce [13, 76]. Out of 37 QTL identified under LN in the present study, two major promising pleiotropic QTL (RM13201-RM13209 and RM13181-RM13201) from the donor (PTB1) and 50 promising RILs with >10 g grain yield with positive QTL were identified (Table 3).

Significant variation was observed for 24 traits/parameters of the present study between the parents and among the RILs across wet and dry seasons corroborating reported genetic and seasonal variability in rice under LN (Table 1). Wide genetic variability under LN has been known in rice based on its sub-species (*indica* vs *japonica*, plant type (land races vs improved), duration (short vs medium vs long) and other factors [35]. Genotype by environment interaction (including the radiation, temperature and soil) was reported in several studies for NUE-QTL mapping studies in rice [27, 77–79]. Under LN, SPAD was reduced significantly in parents and RILs as expected which could be due to the reduced allocation of N to chloroplasts and lower N as reported by Metwally et al. [80]. Decrease of flag leaf length and width, thereby decreasing flag leaf area was also noted under LN as reported in previous studies [80, 81]. Though overall decrease of plant height, number of tillers and productive tillers under LN was observed, the reduction was less in the efficient parent PTB1 which might be due to effective utilization of available N for maintaining the cell size and meristematic activity [82–85]. The reduction in DFF is in agreement with the earlier reports of the low N condition being perceived as stress by the plant, thereby early flowering for the completion of lifecycle under unfavorable conditions [80]. The effect of low N towards reduction on panicle weight, grain yield, test weight and grain harvest index and the yield related traits (PNO, GY, GRNO, TDM, HSW) was well reported in rice [80, 86–88]. The parent (PTB1) with higher N use efficiency has shown buffering capacity to LN expressed in terms of yield and yield related traits and other agro-morphological characters. The other parent BPT5204 also appeared to possess a few positive characters under low N such as leaf width, leaf area. Interestingly, >50 RILs with promising yield (> 10g) under LN were obtained indicating transgressive variants with the positive recombination of characters from both parents (Table 1). The promising lines obtained in present study confirm the feasibility of combining favorable characters resulting in higher yield under LN. As reported, significant genotypic differences were also detected for NDT traits in our study [27]. The use of NUE indices was indicated for identification of high and low NUE aromatic rice genotypes [7], similarly we could identify RILs with promising NUE based on NUE indices for wet and dry seasons. Correlation and path analyses suggested important traits for the purpose of selection under LN for grain yield (Fig 2). In accordance with previous reports, PNUE was found to be correlated negatively with GY under low N suggesting the importance of remobilization in addition to the trait of N uptake [27]. The negative correlation of relative traits *viz*., RGY, RBM, RGN and RBN with NUE parameters suggests that the relative traits could be contradictory for selection.

Identification of markers/QTL associated with traits of interest under LN would be useful in MAS for the development of rice varieties with NUE. Trait wise marker association exclusively under LN through SMA has identified more markers during the dry season over wet season. GNPP (grain nitrogen per plant), TDM (total dry matter) and GY (grain yield) found to be associated with >10 markers which is expected owing to the complexity of the traits (S10 Table). Around 50% of the identified markers in the present study have shown associations with more than five traits/parameters and co-localized with the reported genomic regions. SMA has identified 13 SSRs associated with 10 or more than 10 traits (RM10090, RM1167, RM7075, RM12276, RM13021, RM13197, RM13181, RM3763, RM15855, RM19341, RM7110, RM5455 and RM287). As these 13 SSRs were also co-localized with reported QTL regions for related traits under LN or RN, the regions of these markers are being proposed for their utilization in MAS and also for further characterization.

Selective genotyping approach showed five genomic regions associated with yield under LN reiterating the utility of this approach in saving of time and resources for identification of marker associated genomic regions as reported in earlier studies [89]. Since yield is the key component for NUE, the focus was on identification of genomic regions with yield and yield related traits under LN and their corroboration with the earlier reported genomic regions with yield in rice either under RN or LN. In the present study, 19 QTL on chromosomes 1 and 2 have been identified in common across seasons and treatments out of 37 QTL under LN and 32 QTL under RN. The phenotypic variation of these QTL ranged from 4% to 67% and the contribution of QTL was observed to be from both parents of the RIL. From our study and reported studies, two kinds of QTL were observed *viz*., QTL common for both treatments and QTL found to be exclusive for either LN or RN. The basic mechanism for N metabolism in rice is similar, thus common QTL are expected for LN and RN as reported [19, 90]. However, under LN condition, the plant needs to adapt with additional mechanisms; therefore, different QTL/markers can also be expected as observed in the present study and earlier studies [19]. Pyramiding of these two kinds of QTL would be ideal for development of NUE varieties. Similarly, a few QTL were observed in common across seasons, whereas some exclusive QTL were found either in WS or DS. Thus, the consistent QTL could be deployed or the season specific QTL could be used for need based specific adaptability.

In the present study, we found five genomic regions harboring two or more than 2 QTL. The major stable pleiotropic QTL identified in our study (RM13201—RM13209) from PTB1 spanning 825.4 kb region associated with straw N % (SNP) in both treatments across seasons and yield and yield related traits in WS appears to be promising for the MAS. Out of eight QTL identified in this region, five QTL (DFF, TDM, GY, GRNO and SNP) have shown PV >38%. The second major QTL (RM13181-RM13201) was found to be associated with only relative trait parameters *viz*., RBN, RBM and RGN with more than 58% PV. Wei et al [27] indicated the relationship of NDT and NUE and utility of identified NDT QTL as targets for the development rice cultivars with NUE. Further characterization of two markers (RM13181 and RM13209) from the two major QTL has shown the positive average allele effect (same allele as PTB1) with grain yield in 54 genotypes under LN strengthening the confidence of identified QTL in the present study.

After the first QTL study under LN in rice [14], more than 200 QTL have been reported for differential N in rice [13, 76], however their use in the development of NUE cultivars is less reported owing to the need of validation of reported QTL, major QTL with higher values of PV and the availability of donor parent in which QTL were identified. More than half of the QTL identified in the present study are major QTL with PV >30% and five of the identified QTL were pleiotropic The QTL with multiple effects or QTL hotspots/QTL clusters under LN have also been reported earlier [23, 22, 91]. Several proteins and transporter genes identified within the QTL are being further studied.

## Conclusion

Grain yield and nitrogen use efficiency are complex traits and depend on interaction of various primary traits. Correlation and path coefficient analysis of the present study suggests that PH, TNO, PNO, GRNO, TDM, and GNPP need to be considered for the rice yield improvement under LN. More than 50 promising RIL >10 g yield under LN were identified. Using single marker analysis, 144 marker trait associations were noted under LN, out of which 13 markers could be deployed either for MAS or for fine mapping. A major stable pleiotropic (RM13201—RM13209) from PTB1 region associated with straw N % (SNP) in both treatments across seasons and yield and yield related traits in WS and another major QTL (RM13181-RM13201) associated with only relative trait parameters of biomass, grain and grain nitrogen were identified for utilization in marker assisted breeding programs and further characterization.

## Supporting information

S1 Table Screening of 107 rice genotypes under low and recommended N field conditions. WS: Wet season, DS: Dry season, Low N: Low nitrogen, Rec N: Recommended nitrogen.
(XLSX)

S2 Table A. Details of soil properties of the experimental plot. B. Details of weather parameters during the experiement.
(XLSX)

S3 Table Details of 54 genotypes and parents screend under LN and RN during 2014 (WS) and 2015 (DS). WS: Wet season, DS: Dry season, P: Parent.
(XLSX)

S4 Table Details of SSRs used for parental polymorphism survey, polymorphic between the parents, for selective genotyping, for local linkage map and total polymorphic. (XLSX)

S5 Table Details of analysis of variance for parents (BPT5204 and PTB1). WS: Wet season, DS: Dry season.
(XLSX)

S6 Table Percentage reduction in morpho-physiological, agronomic and yield traits of parents and RILs in wet and dry seasons. SPAD: SPAD value, LL: leaf length (cm), LW: leaf width (cm), LA: leaf area (cm), DFF: days to 50% flowering, PH: plant height (cm), TNO: tiller number, PNO: panicle number: GRNO: grain number, HSW: hundred seed weight (g), GY: grain yield per plant (g/plant), TDM: total dry matter (g/plant), GNP: grain nitrogen percent, SNP: straw nitrogen percent, GNPP: grain nitrogen/ plant, SNPP: straw nitrogen/ plant.
(XLSX)

S7 Table A. Path coefficient analysis of morpho physiological, agronomic and yield traits under low nitrogen in wet season. SPAD: SPAD value, LL: Leaf length (cm), LW: Leaf width (cm), LA: Leaf area (cm), DFF: Days to 50% flowering, PH: Plant height (cm), TNO: Tiller number, PNO: Panicle number: GRNO: Grain number, HSW: Hundred seed weight (g), GY: Grain yield per plant (g/plant), TDM: Total dry matter (g/plant), GNP: Grain nitrogen percent, SNP: Straw nitrogen percent, GNPP: Grain nitrogen/ plant, SNPP: Straw nitrogen/ plant, NHI: Nitrogen harvest index. B. Path coefficient analysis of morpho physiological, agronomic and yield traits under low nitrogen in dry season. SPAD: SPAD value, LL: Leaf length (cm), LW: Leaf width (cm), LA: Leaf area (cm), DFF: Days to 50% flowering, PH: Plant height (cm), TNO: Tiller number, PNO: Panicle number: GRNO: Grain number, HSW: Hundred seed weight (g), GY: Grain yield per plant (g/plant), TDM: Total dry matter (g/plant), GNP: Grain nitrogen percent, SNP: Straw nitrogen percent, GNPP: Grain nitrogen/ plant, SNPP: Straw nitrogen/ plant, NHI: Nitrogen harvest index. C. Path coefficient analysis of morpho physiological, agronomic and yield traits under recommended nitrogen in wet season. SPAD: SPAD value, LL: Leaf length (cm), LW: Leaf width (cm), LA: Leaf area (cm), DFF: Days to 50% flowering, PH: Plant height (cm), TNO: Tiller number, PNO: Panicle number: GRNO: Grain number, HSW: Hundred seed weight (g), GY: Grain yield per plant (g/plant), TDM: Total dry matter (g/plant), GNP: Grain nitrogen percent, SNP: Straw nitrogen percent, GNPP: Grain nitrogen/ plant, SNPP: Straw nitrogen/ plant, NHI: Nitrogen harvest index. D. Path coefficient analysis of morpho physiological, agronomic and yield traits under recommended nitrogen in dry season. SPAD: SPAD value, LL: Leaf length (cm), LW: Leaf width (cm), LA: Leaf area (cm), DFF: Days to 50% flowering, PH: Plant height (cm), TNO: Tiller number, PNO: Panicle number: GRNO: Grain number, HSW: Hundred seed weight (g), GY: Grain yield per plant (g/plant), TDM: Total dry matter (g/plant), GNP: Grain nitrogen percent, SNP: Straw nitrogen percent, GNPP: Grain nitrogen/ plant, SNPP: Straw nitrogen/ plant, NHI: Nitrogen harvest index.
(XLSX)

S8 Table Segregation table of associated SSRs with grain yield under LN by selective genotyping (MapDisto v. 1.7 software (Lorieux 2007)). (XLSX)

S9 Table List of polymorphic SSRs between parents BPT5204 and PTB1identified from the saturation of yield associated genomic regions. (XLSX)

S10 Table A. Trait wise marker association under low nitrogen identified through single marker analysis. SPAD: SPAD value, LL: leaf length (cm), LW: leaf width (cm), LA: leaf area (cm), DFF: days to 50% flowering, PH: plant height (cm), TNO: tiller number, PNO: panicle number: GRNO: grain number, HSW: hundred seed weight (g), GY: grain yield per plant (g/plant), TDM: total dry matter (g/plant), GNP: grain nitrogen percent, SNP: straw nitrogen percent, GNPP: grain nitrogen/ plant, SNPP: straw nitrogen/ plant, NHI: nitrogen harvest index, RGY: relative grain yield, RBM: relative biomass yield, RGN: relative grain nitrogen, RBN: relative biomass nitrogen, PNUE: physiological nitrogen use efficiency, ANUE: agronomic nitrogen use efficiency, APE: agro- physiological efficiency. B. Chromosome wise marker trait association under low nitrogen identified through single marker analysis. SPAD: SPAD value, LL: leaf length (cm), LW: leaf width (cm), LA: leaf area (cm), DFF: days to 50% flowering, PH: plant height (cm), TNO: tiller number, PNO: panicle number: GRNO: grain number, HSW: hundred seed weight (g), GY: grain yield per plant (g/plant), TDM: total dry matter (g/plant), GNP: grain nitrogen percent, SNP: straw nitrogen percent, GNPP: grain nitrogen/ plant, SNPP: straw nitrogen/ plant, NHI: nitrogen harvest index, RGY: relative grain yield, RBM: relative biomass yield, RGN: relative grain nitrogen, RBN: relative biomass nitrogen, PNUE: physiological nitrogen use efficiency, ANUE: agronomic nitrogen use efficiency, APE: agro- physiological efficiency.
(XLSX)

S11 Table A. Trait wise associations with phenotypic traits with single marker analysis of for nine polymorphic SSRs. SPAD: SPAD value, LL: leaf length (cm), LW: leaf width (cm), LA: leaf area (cm), DFF: days to 50% flowering, PH: plant height (cm), TNO: tiller number, PNO: panicle number: GRNO: grain number, HSW: hundred seed weight (g), GY: grain yield per plant (g/plant), TDM: total dry matter (g/plant), GNP: grain nitrogen percent, SNP: straw nitrogen percent, GNPP: grain nitrogen/ plant, SNPP: straw nitrogen/ plant, NHI: nitrogen harvest index, RGY: relative grain yield, RBM: relative biomass yield, RGN: relative grain nitrogen, RBN: relative biomass nitrogen, PNUE: physiological nitrogen use efficiency, ANUE: agronomic nitrogen use efficiency, APE: agro- physiological efficiency. B. Marker wise associations with phenotypic traits with single marker analysis of for nine polymorphic SSRs. SPAD: SPAD value, LL: leaf length (cm), LW: leaf width (cm), LA: leaf area (cm), DFF: days to 50% flowering, PH: plant height (cm), TNO: tiller number, PNO: panicle number: GRNO: grain number, HSW: hundred seed weight (g), GY: grain yield per plant (g/plant), TDM: total dry matter (g/plant), GNP: grain nitrogen percent, SNP: straw nitrogen percent, GNPP: grain nitrogen/ plant, SNPP: straw nitrogen/ plant, NHI: nitrogen harvest index, RGY: relative grain yield, RBM: relative biomass yield, RGN: relative grain nitrogen, RBN: relative biomass nitrogen, PNUE: physiological nitrogen use efficiency, ANUE: agronomic nitrogen use efficiency, APE: agro- physiological efficiency.
(XLSX)

S12 Table Details of QTL identified through CIM for traits under Recommeneded Nitrogen (RN). SPAD: SPAD value, LL: leaf length (cm), LW: leaf width (cm), LA: leaf area (cm), DFF: days to 50% flowering, PH: plant height (cm), TNO: tiller number, PNO: panicle number: GRNO: grain number, HSW: hundred seed weight (g), GY: grain yield per plant (g/plant), TDM: total dry matter (g/plant), GNP: grain nitrogen percent, SNP: straw nitrogen percent, GNPP: grain nitrogen/ plant, SNPP: straw nitrogen/ plant, NHI: nitrogen harvest index, RN: Recommended nitrogen, LN: Low nitrogen, WS: Wet season, DS: Dry season.
(XLSX)

S13 Table The details of putative candidate genes in the region of pleiotropic QTL. (XLSX)

S1 Fig Details of inter trait correlation under recommended nitrogen in wet season (1-A), recommended nitrogen in dry season (1-B), nitrogen efficiency indices in wet season (1-C) and nitrogen efficiency indices in dry season (1-D). SPAD: SPAD value, LL: leaf length (cm), LW: leaf width (cm), LA: leaf area (cm), DFF: days to 50% flowering, PH: plant height (cm), TNO: tiller number, PNO: panicle number: GRNO: grain number, HSW: hundred seed weight (g), GY: grain yield per plant (g/plant), TDM: total dry matter (g/plant), GNP: grain nitrogen percent, SNP: straw nitrogen percent, GNPP: grain nitrogen/ plant, SNPP: straw nitrogen/ plant, NHI: nitrogen harvest index, RGY: relative grain yield, RBM: relative biomass yield, RGN: relative grain nitrogen, RBN: relative biomass nitrogen, PNUE: physiological nitrogen use efficiency, ANUE: agronomic nitrogen use efficiency, APE: agro physiological efficiency.
(DOCX)

S2 Fig Venn diagram showing unique and shared marker trait associations across under low and recommended nitrogen levels across wet and dry seasons. (DOC)
