# Supplementary material for: A major pleiotropic QTL identified for yield components and nitrogen content in rice (Oryza sativa L.) under differential nitrogen field conditions
Source: PLoS One. 2020 Oct 20;15(10):e0240854. doi: 10.1371/journal.pone.0240854 (PMC7575116; doi:10.1371/journal.pone.0240854)
Supplement: S1 Fig — Details of inter trait correlation under recommended nitrogen in wet season (1-A), recommended nitrogen in dry season (1-B), nitrogen efficiency indices in wet season (1-C) and nitrogen efficiency indices in dry season (1-D). SPAD: SPAD value, LL: leaf length (cm), LW: leaf width (cm), LA: leaf area (cm), DFF: days to 50% flowering, PH: plant height (cm), TNO: tiller number, PNO: panicle number: GRNO: grain number, HSW: hundred seed weight (g), GY: grain yield per plant (g/plant), TDM: total dry matter (g/plant), GNP: grain nitrogen percent, SNP: straw nitrogen percent, GNPP: grain nitrogen/ plant, SNPP: straw nitrogen/ plant, NHI: nitrogen harvest index, RGY: relative grain yield, RBM: relative biomass yield, RGN: relative grain nitrogen, RBN: relative biomass nitrogen, PNUE: physiological nitrogen use efficiency, ANUE: agronomic nitrogen use efficiency, APE: agro physiological efficiency. (DOCX) [file pone.0240854.s014.docx]

**S1 Fig.** Details of inter trait correlation under recommended nitrogen in wet season (1-A), recommended nitrogen in dry season (1-B), nitrogen use efficiency indices in wet season (1-C) and nitrogen use efficiency indices in dry season (1-D)

| 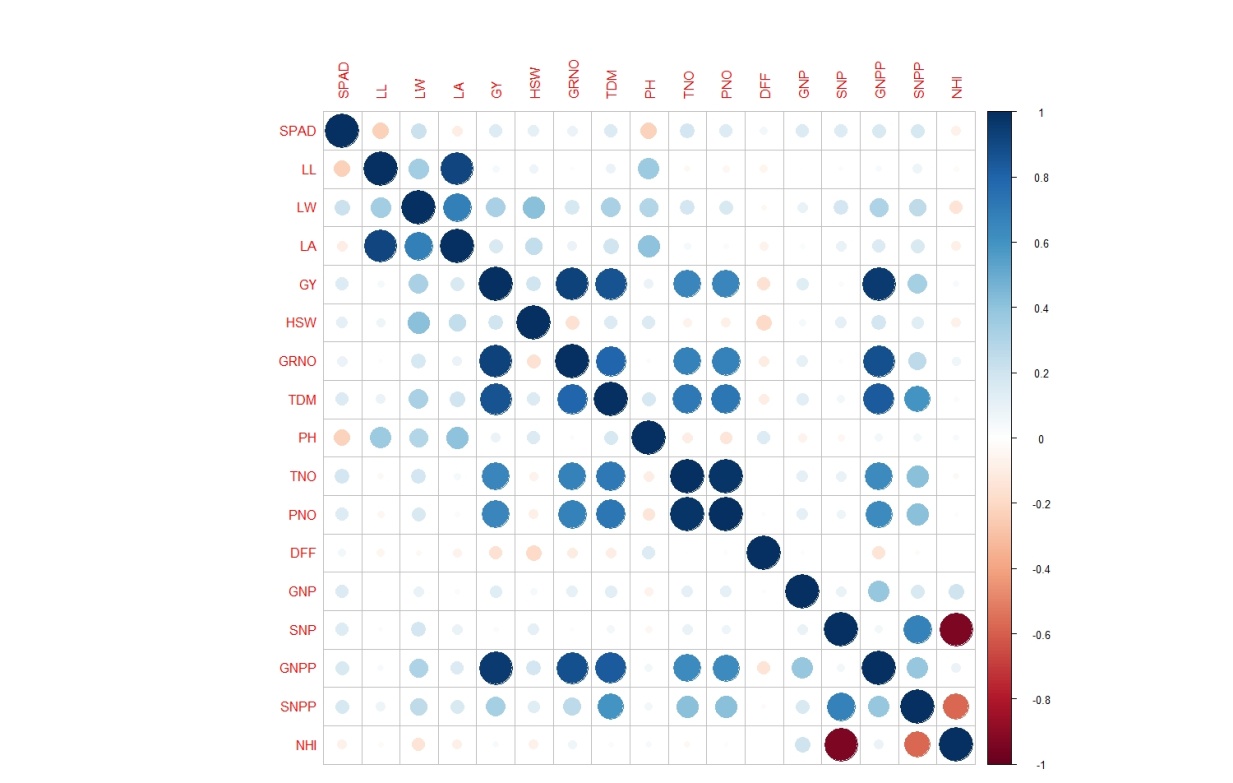  1-A |
| --- |
| 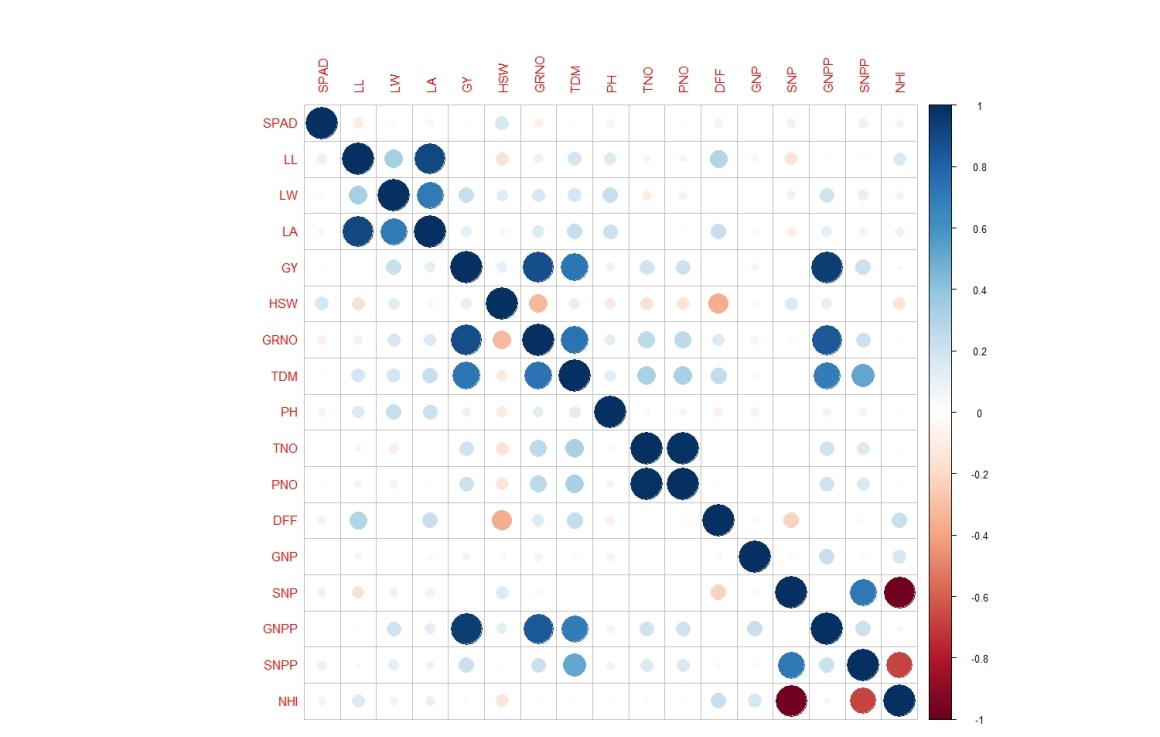  1-B |
| 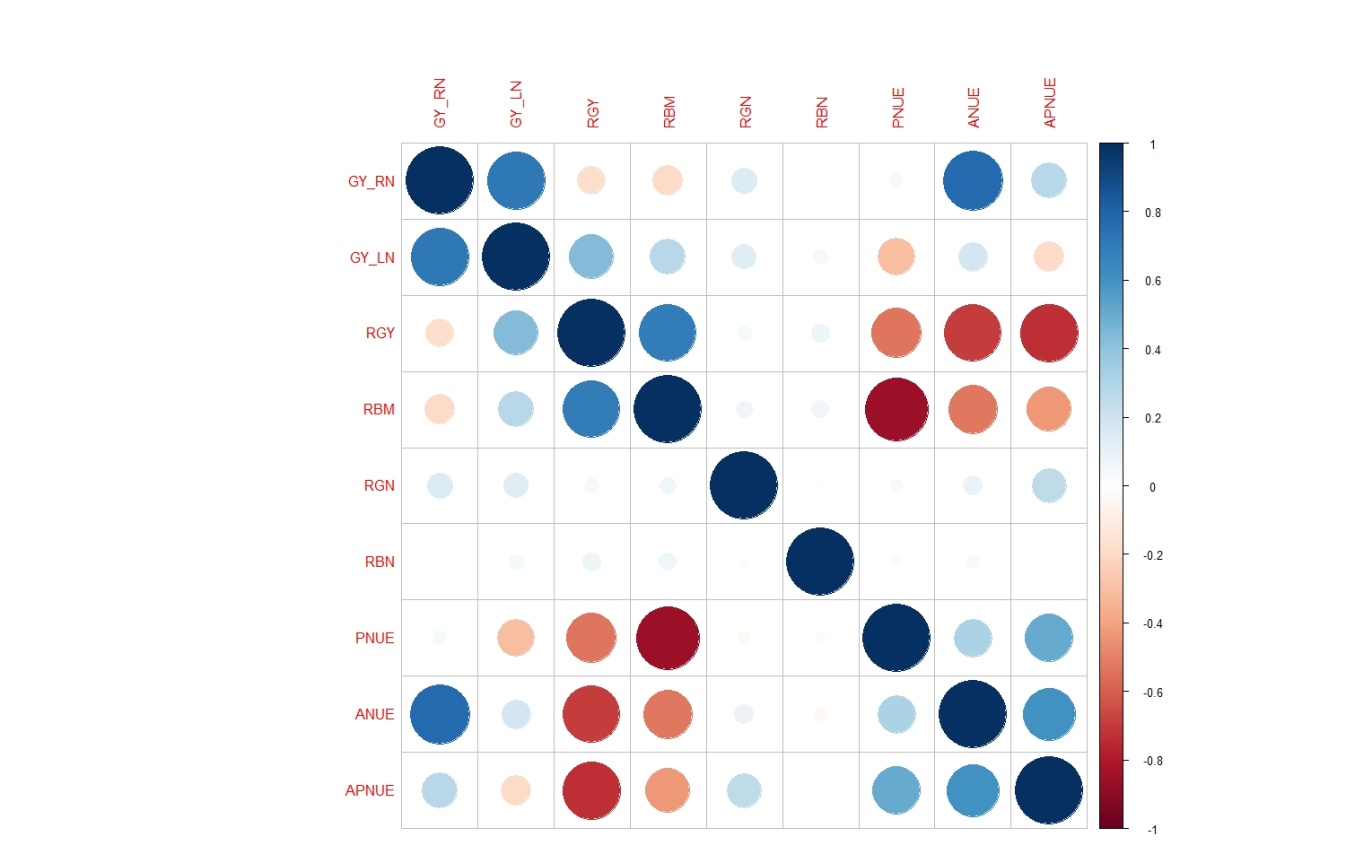  1-C |
| 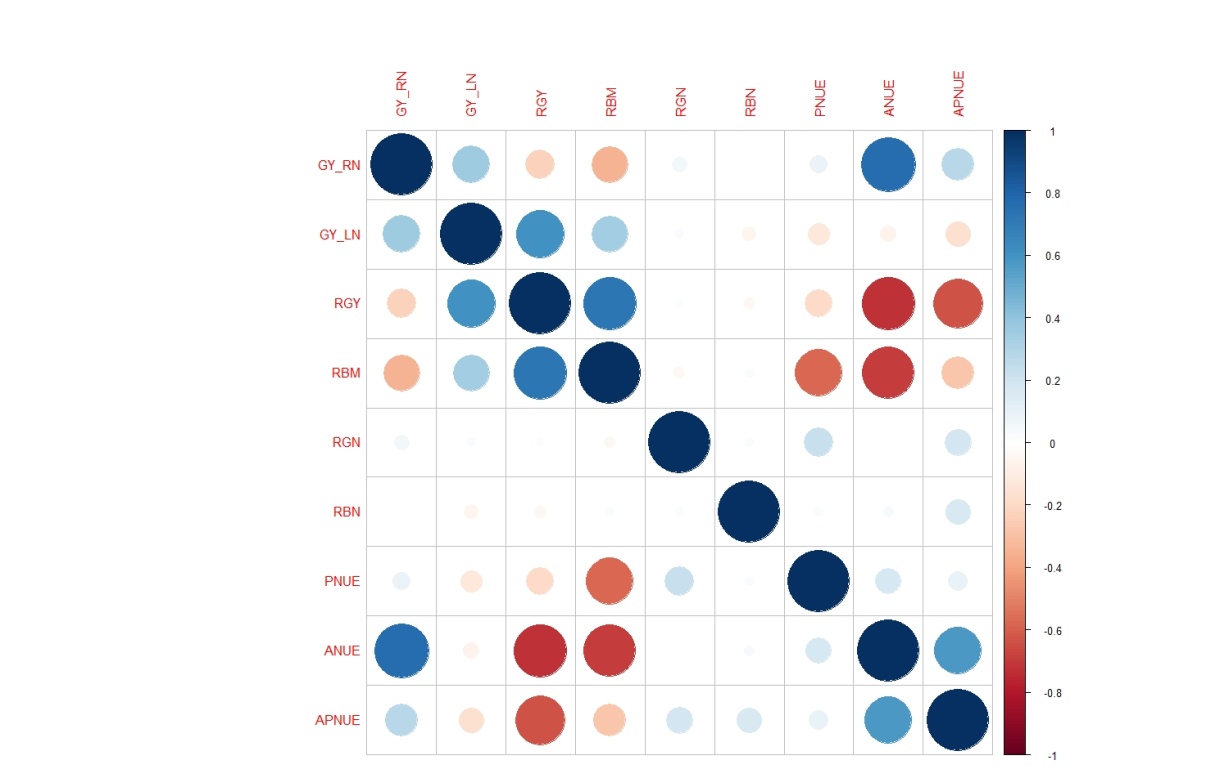  1-D |

SPAD: SPAD value, LL: leaf length (cm), LW: leaf width (cm), LA: leaf area (cm), DFF: days to 50% flowering, PH: plant height (cm), TNO: tiller number, PNO: panicle number: GRNO: grain number, HSW: hundred seed weight (g), GY: grain yield per plant (g/plant), TDM: total dry matter (g/plant), GNP: grain nitrogen percent, SNP: straw nitrogen percent, GNPP: grain nitrogen/ plant, SNPP: straw nitrogen/ plant, NHI: nitrogen harvest index, RGY: relative grain yield, RBM: relative biomass yield, RGN: relative grain nitrogen, RBN: relative biomass nitrogen, PNUE: physiological nitrogen use efficiency, ANUE: agronomic nitrogen use efficiency, APE: agro-physiological efficiency
