# Supplementary material for: A major pleiotropic QTL identified for yield components and nitrogen content in rice (Oryza sativa L.) under differential nitrogen field conditions
Source: PLoS One. 2020 Oct 20;15(10):e0240854. doi: 10.1371/journal.pone.0240854 (PMC7575116; doi:10.1371/journal.pone.0240854)

S2 Fig. Venn diagram showing unique and shared marker trait associations across under low and recommended nitrogen levels across wet and dry seasons.


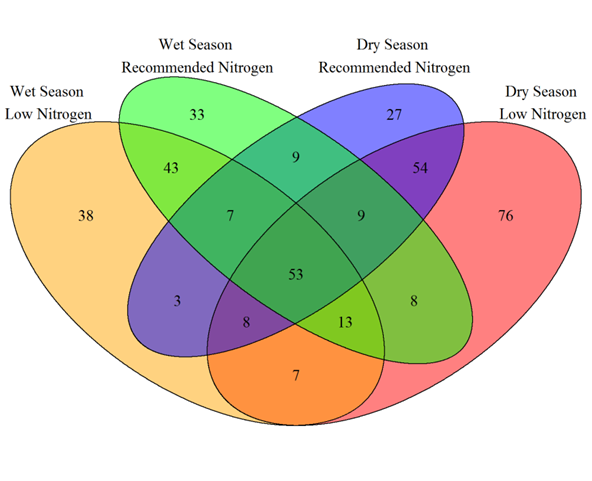

Supplement: S2 Fig — (DOC) [file pone.0240854.s015.doc]
